# Pt-Bi decorated nanoporous gold for high performance direct glucose fuel cell

**DOI:** 10.1038/srep39162

**Published:** 2016-12-14

**Authors:** Hong Guo, Huiming Yin, Xiuling Yan, Shuai Shi, Qingyang Yu, Zhen Cao, Jian Li

**Affiliations:** 1 Tianjin Key Laboratory of Advanced Functional Porous Materials and Institute for New Energy Materials and Low Carbon Technologies, School of Materials Science and Engineering, Tianjin University of Technology, Tianjin 300384, China; 2 School of Chemistry and Environmental Science, Yili Normal University, Xinjiang 835000, China

## Abstract

Binary PtBi decorated nanoporous gold (NPG-PtBi) electrocatalyst is specially designed and prepared for the anode in direct glucose fuel cells (DGFCs). By using electroless and electrochemical plating methods, a dense Pt layer and scattered Bi particles are sequentially coated on NPG. A simple DGFC with NPG-PtBi as anode and commercial Pt/C as cathode is constructed and operated to study the effect of operating temperatures and concentrations of glucose and NaOH. With an anode noble metal loading of only 0.45 mg cm^−2^ (Au 0.3 mg and Pt 0.15 mg), an open circuit voltage (OCV) of 0.9 V is obtained with a maximum power density of 8 mW cm^−2^. Furthermore, the maximum gravimetric power density of NPG-PtBi is 18 mW mg^−1^, about 4.5 times higher than that of commercial Pt/C.

Finding an alternative to environmentally unfriendly energy resources from fossil fuels is becoming an urgent task owing to the increasing environmental pollution and energy shortage^1^,^2^,^3^,^4^,^5^. Therefore, studies focusing on exploring pollution-free energy production, conversion and storage devices have attracted a large number of investigators^6^,^7^,^8^. With the features of nontoxic, non-flammable, high energy density and abundant resource of glucose, direct glucose fuel cell (DGFC) is a promising energy conversion device^9^,^10^,^11^,^12^. Typically, in DGFC the electric energy is generated by electrocatalyzing complementary oxidation and reduction reactions at a couple of corresponding anode and cathode, respectively^13^,^14^,^15^. Thus, high efficient anode is vital important and dependent on the types of DGFCs. Recently, plenty of researches have been focused on microbial and enzymatic glucose oxidizing catalysts^16^,^17^,^18^,^19^,^20^. However, the complicated operation conditions and shortcomings of biotic catalysts have impeded their widely application^21^. To overcome the drawbacks of microbial or enzymatic fuel cells, abiotic glucose fuel cells have been explored^22^,^23^.

The studies in the past few decades on electro-oxidation of sugars show that noble metal catalysts such as Pt, Pd and Au could potentially oxidize monosaccharides such as glucose, mannose and fructose^24^,^25^,^26^,^27^,^28^,^29^. Among various metallic materials, Pt has been extensively investigated for its high activity toward glucose oxidation^13^. Unfortunately, its active sites are so easily poisoned by unexpected intermediates such as CO causing performance deterioration^30^. Therefore, bimetallic electrocatalysts, such as PtRu, PtAu, PtPd, PtBi are developed to reduce the poisoning of platinum surface, which can suppress adsorption of poisoning intermediates^31^,^32^,^33^,^34^. Among these choices, Bismuth wins more opportunities due to its cost advantage^31^,^35^,^36^. Debika Basu *et al*. synthesized PtBi/C catalyst by immobilizing metal sols on carbon substrate and utilized this as anode for direct glucose alkaline fuel cell with Nafion^®^ membrane^32^. The maximum gravimetric power density is 1.25 mW cm^−2^. They also synthesized PtAu/C anode for DGFC which exhibited a maximum gravimetric power density of 1.6 mW cm^−2^ in comparison with commercial PtRu/C (1.13 mW cm^−2^)^32^. Cheng Chuan Chen *et al*.^10^ prepared Pd-Bi/C electrocatalyst by one-pot polyol method and the maximum power density is 1.42 mW cm^−2^.

Nanoporous metals, with suitable bicontinuous ligament/pore structure for mass transfer and electron conduction, have demonstrated good applicability for electrode materials^37^,^38^,^39^. Typically, nanoporous gold (NPG) has been adopted in anode for directly organic molecules based fuel cells, e.g. methanol, formic acid^40^,^41^. Besides the good catalytic activity of NPG in glucose, hydrazine hydrate electrooxidation reactions and etc^42^,^43^,^44^, electrocatalysts by plating NPG substrate with Pt, Pd *et al*. also exhibit outstanding catalytic activities^45^,^46^,^47^.

In this work, by using bicontinuous NPG as the substrate, a binary PtBi electrocatalyst (NPG-PtBi) was specially designed and facilely fabricated for glucose oxidation reaction (GOR). After the electrochemical tests towards GOR by using cyclic voltammetry, the optimized NPG-PtBi electrocatalyst was chosen for anode in DGFC. Single cells composed of an anode of NPG-PtBi, a cathode of commercial Pt/C were constructed, and the effects of pH values, fuel and electrolyte concentrations, and operation temperatures were systematically investigated.

## Results

### Morphological and compositional characterization of NPG-PtBi

**Figure 1:**
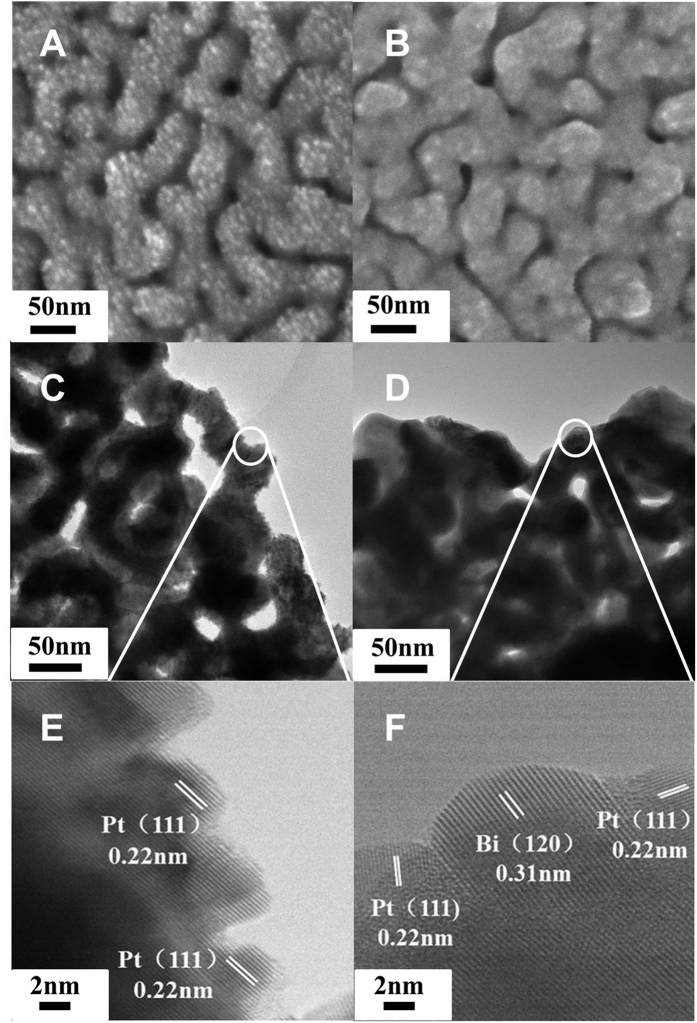
SEM (**A**,**B**), TEM (**C**,**D**) and HRTEM (**E**,**F**) images of NPG-Pt (**A**,**C**,**E**), NPG-PtBi (**B**,**D**,**F**).

The scanning electron microscope (SEM) and transmission electron microscope (TEM) images of Pt decorated nanoporous gold (NPG-Pt) (Fig. 1A and C) demonstrate interconnected ligaments with size about 20 nm. As shown in Fig. 1A and C, the entire ligament surface is uniformly covered by a ~2 nm thick layer of nanoparticles. As the ligament of pristine NPG is characterized by a smooth surface^39^, these small nanoparticles can be ascribed to Pt, which is evidenced by the lattice space 0.22 nm corresponding to the (111) facets of Pt (Fig. 1E). The well-resolved, highly ordered lattice fringes extending to NPG substrate demonstrate that Pt nanoparticles are grown on the surface of NPG in an epitaxial mode which is consistent with our previous results^48^. Figure 1B and D show the SEM and TEM images of NPG-PtBi. As can be seen from Fig. 1B, Pt islands become unclear after electrochemical deposition of Bi specie. Measurement of the ordered lattice fringes (Fig. 1F) indicates that the surface of NPG-PtBi is covered by both Pt and Bi species. Attributed to the close lattice spaces (0.328 and 0.326 nm) of Bi (0 1 2) and Bi_2_O_3_ (1 2 0), the measured data (0.31 nm) of Bi specie cannot be accurately described.

**Figure 2:**
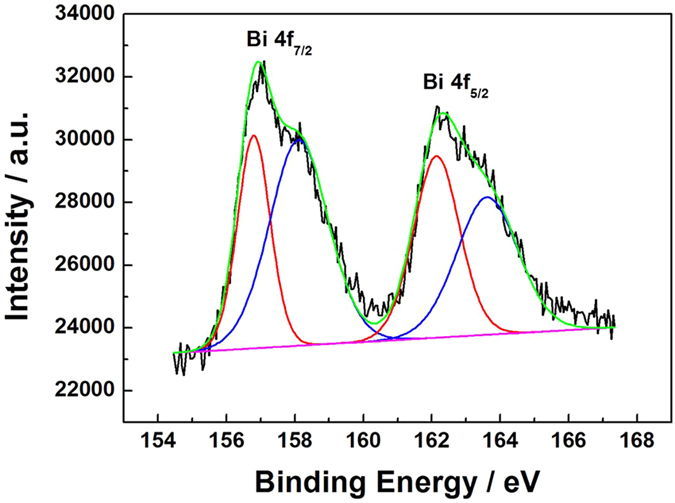
XPS spectrum of NPG-PtBi for Bi 4 f core.

Considering that X-ray photoelectron spectroscopy (XPS) is more sensitive to the surface electron structure, XPS spectra towards Bi 4 f core-level in NPG-PtBi was measured, which presented two pairs of peaks (in Fig. 2). According to previous works^49^,^50^, the doublet signals with binding energies of 157 and 162 eV can be assigned to Bi 4f_7/2_ and 4f_5/2_ for metallic Bi, while the other doublet signals with binding energies of 158.3 and 163.5 are ascribed to the oxidized state of Bi in Bi_2_O_3_. Therefore, NPG-PtBi was constructed with a Bi and Bi_2_O_3_ co-decorated Pt layer on NPG substrate. In the electrocatalytical oxidation reaction, Bi^0^ and Bi^3+^ play a synergistic action. In alkaline solution, Bi^0^ atoms trend to absorb more OH^−^ to form hydroxyl which may be shared by the below adjacent Pt atom. Bi^3+^ ion exist in various states such as Bi(OH)_3_, BiOOH in alkaline solution. Similarly, the hydroxyl is shared by the neighboring Pt, resulting in the increased electrocatalysis^31^.

In addition, each NPG-Pt sample used in this work possesses Au and Pt loadings of approximately 0.1 and 0.05 mg cm^−2^, respectively, conducted by energy-dispersive spectrometry (EDS). By using inductive coupled plasma optical emission spectrometry (ICP-OES), Au and Pt loadings of 0.11 and 0.054 mg cm^−2^ was obtained, which is similar to the results from EDS.

### Electrochemical performance of NPG-PtBi

**Figure 3:**
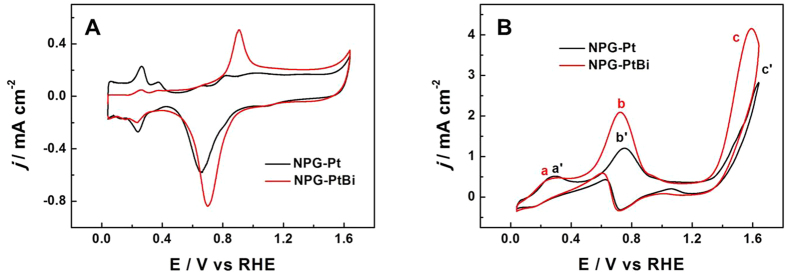
CV curves of NPG-Pt and NPG-PtBi in (**A**) 0.1 M NaOH and (**B**) mixture solution of 0.1 M NaOH and 10 mM glucose. The current densities were normalized to the geometric area of the electrode (0.1256 cm^2^). Sweep rate: 20 mV s^−1^.

Electrocatalytic properties of NPG-PtBi were measured by cyclic voltammetry (CV). In addition, potentials given in this paper all were versus reversible hydrogen electrode (RHE). To reveal the enhancement from Bi specie toward GOR, electrocatalytic activity of NPG-PtBi was estimated relative to NPG-Pt. Figure 3A and B separately exhibited the CV curves in 0.1 M NaOH solution (Fig. 3A) and mixture solution of 0.1 M NaOH and 10 mM glucose (Fig. 3B). In Fig. 3A, the disappeared signals for Au oxide formation and reduction indicate a completely covered NPG surface by Pt specie towards NPG-Pt^42^. Connecting with the morphology and composition of NPG-Pt, it should be noted that there is negligible effect of NPG substrate on electrocatalytic activity of surface PtBi. Consulting previous literatures, electrocatalytic surface area (ECSA) of Pt could be measured by integrating the faradaic charges (Q_H_) associated with the hydrogen adsorption/desorption via the equation: ECSA = Q_H_/(210 μC cm^−2^)^40^. Due to the fact that hydrogen does not adsorb on Bi specie, CV curve of NPG-PtBi in Fig. 3A demonstrates that Pt surface is partly covered by Bi specie as expected, because of the obviously decreased integrated areas of hydrogen adsorption/desorption peaks. In addition, the apparently increased oxidation at about 0.9 V and reduction peaks at 0.7 V of NPG-PtBi could be generally ascribed to the superposition of oxides formation and reduction of Pt and Bi^31^.

In contrast to the reduced surface Pt atoms, CV curves of NPG-Pt and NPG-PtBi in alkaline glucose solution (Fig. 3B) demonstrate that NPG-PtBi shows an even higher catalytic activity towards GOR. According to Fig. 3B, during the forward potential scanning, peaks (a and a’) at about 0.3 V are assigned to the dehydrogenated adsorption of glucose both on NPG-Pt and NPG-PtBi surfaces, which are generally equal to each other. Peaks (b and b’) at ~0.7 V are ascribed to the further oxidation of the previously generated intermediates^31^. Peaks (c and c’) at ~1.6 V, are often attributed to the further oxidation of gluconate to 2-keto-gluconate with 4e transferring, a characteristic catalytic reaction for Bi^31^. According to the CV curve of NPG-PtBi, an obviously negative shift, about 35 mV of the peak current potential (peak b’) exhibits in comparison to that of NPG-Pt (peak b), together with ~2 times higher peak current density.

**Figure 4:**
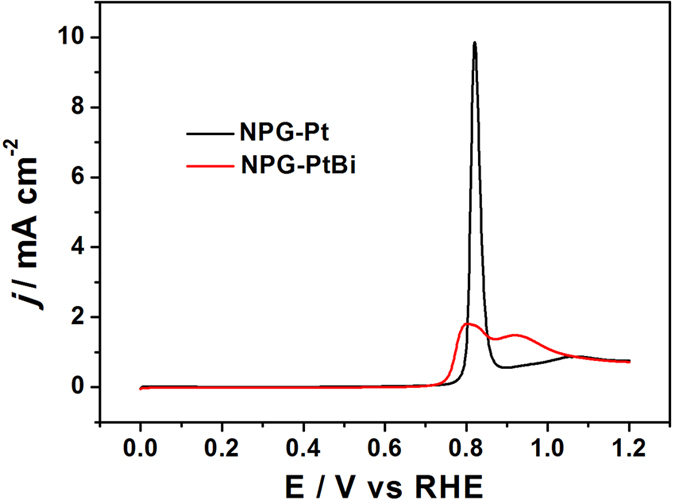
CO stripping curves of NPG-Pt and NPG-PtBi in 0.5 M H_2_SO_4_. Sweep rate: 50 mV s^−1^.

To find out the possible reasons for the activity increase of NPG-PtBi relative to NPG-Pt, CO stripping curves were obtained by exposing the NPG-Pt and NPG-PtBi electrodes to CO saturated 0.5 M H_2_SO_4_ to result in the rapid and irreversible adsorption of CO, which could be monitored by electrochemical oxidation to CO_2_. Stripping CV curves of CO adsorbed NPG-PtBi and unmodified NPG-Pt electrocatalysts are shown in Fig. 4, following saturation of the catalyst surface with adsorbed CO. The potential of CO stripping on NPG-Pt is at 0.82 V, while the CO stripping CV for NPG-PtBi shows a dual peak, with the CO stripping peak potential at 0.79 V, ~30 mV lower than unmodified NPG-Pt^51^. The potential of the second peak at 0.93 V is ascribed to the oxidation of Bi adatoms^51^. Generally, the negative shift of the CO stripping peak is related to the decrease of Pt-CO_ads_ bond strength^52^.

Therefore, Bi specie on surface of NPG-Pt possibly plays two roles. One is the electronic effect of Bi adatoms that cause weakening of CO adsorption due to lower d-band center of Pt^52^. The other is that, more OH^−^ will be adsorbed from the alkaline solution to form hydroxyl ascribed to Bi adatoms, hence increasing the amount of hydroxyl around Pt atoms^35^,^49^. It is generally accepted that Pt-OH sites are active species for the electrochemical oxidation of glucose at negative potentials, which reasonably explains the obviously increased electrocatalytical activity of Bi^0^-Pt at about 0.7 V.

**Figure 5:**
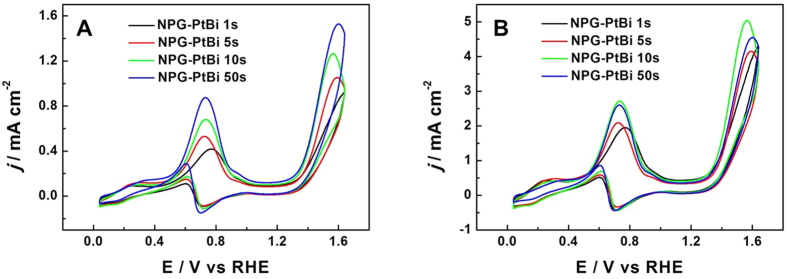
CV curves of NPG-PtBi with various Bi loadings in mixture solution of 0.1 M NaOH and 10 mM glucose separately normalized to (**A**) ECSAs and (**B**) geometric area (0.1256 cm^2^). Sweep rate: 20 mV s^−1^.

Aiming at the requirement of DGFC for higher output power density, anode electrocatalyst with high catalytic activity is selected by comparing the peak current density normalized to geometric areas. To optimize the catalytic activity of NPG-PtBi towards GOR, amounts of Bi component were adjusted by controlling the deposition times. CV curves in mixture solution of 0.1 M NaOH and 10 mM glucose are exhibited in Fig. 5 with the catalytic activity separately normalized to each ECSA (Fig. 5A) and geometric area (Fig. 5B). Generally, a CV curve normalized to its own ECSA indicates the electrocatalyst’s intrinsic catalytic activity. Therefore, according to Fig. 5A the intrinsic electrocatalytic activity of NPG-PtBi is shooting up with the increasing Bi loadings, given by the raising current density of the oxidation peak of glucose at 0.7 V. Figure 5B exhibits that the optimized electrocatalyst for DGFC is the NPG-PtBi sample by electrochemically depositing Bi specie for 10 s, possibly due to the promoted intrinsic activity but reduced surface Pt atoms. Therefore, this electrocatalyst was chosen as the anode in DGFCs in this paper.

### Single fuel cell assembly and performance testing

Single fuel cell of DGFC was constructed and performed by using a NPG-PtBi anode, a commercial Pt/C cathode, and a piece of electrolyte membrane Nafion^®^ 115. Similar to hydrazine-hydrogen peroxide fuel cell^43^, Na^+^ is the main charge carrier during DGFC operation, the following electrode reactions occur at the anode and cathode:


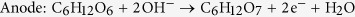



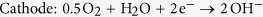


Owing to the seriously low Pt loading on NPG-PtBi (0.05 mg cm^−2^), the anode in DGFC is prepared by attaching 3 layers NPG-PtBi films, with a total Pt loading of 0.15 mg cm^−2^ and a total novel metal (Au and Pt) loading of 0.45 mg cm^−2^. While the single cell was operated, the anodic fuel solution was fed into the anode flow channel by a peristaltic pump with the flow rate of 2.0 mL min^−1^ and oxygen gas was fed to the cathode through a mass flow controller with the flow rate of 100 standard cubic centimeters per minute (SCCM). The effects of pH values, cell temperatures, and concentrations of fuel and electrolyte on the performance of DGFC have been systematically investigated.

**Figure 6:**
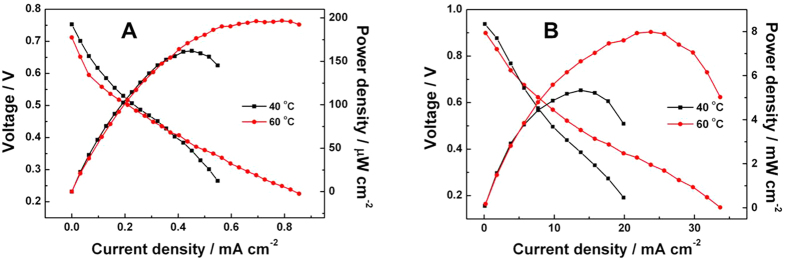
Single-cell performances of NPG-PtBi and Pt/C based MEAs. C-V and C-P polarization curves of DGFC in (**A**) PBS (pH = 7) and (**B**) 2 M NaOH with 0.5 M glucose. The anode NPG-PtBi loaded 0.15 mg_Pt_ cm^−2^ and 0.45 mg_Pt+Au_ cm^−2^, while the commercial Pt/C was used as cathode with Pt loading of 2 mg cm^−2^.

The polarization and power density curves in 0.1 M phosphate buffer solution (PBS, pH = 7) and 2 M NaOH solution are presented in Fig. 6A and B, respectively. The experiments were separately carried out at 40 and 60 °C with 0.5 M glucose solution. The open circuit voltages (OCV) obtained are close to 0.75 and 0.95 V, while the peak power densities are calculated to be 0.2 mW cm^−2^ and 8 mW cm^−2^ separately using 0.1 M PBS and 2 M NaOH solutions. These experimental data indicate that the performance of glucose fuel cell is preferred in alkaline rather than neutral media. This result is consistent with the previous finding that, the rate of electro-oxidation of glucose in alkaline environments is higher than that in neutral media^47^. Nevertheless, the peak power density of the cell with neutral media is higher than that of previous reports, which also suggests NPG-PtBi a promising catalyst for glucose fuel cell in certain environment condition such as living organism^24^. Considering the operation temperatures, 40 °C and 60 °C are selected for comparison to avoid glucose decomposition at higher temperature. As expected, Fig. 6B indicates the fuel cell operated at 60 °C outputs higher power density. So 60 °C was selected as the operation temperature to investigate effects of other factors.

**Figure 7:**
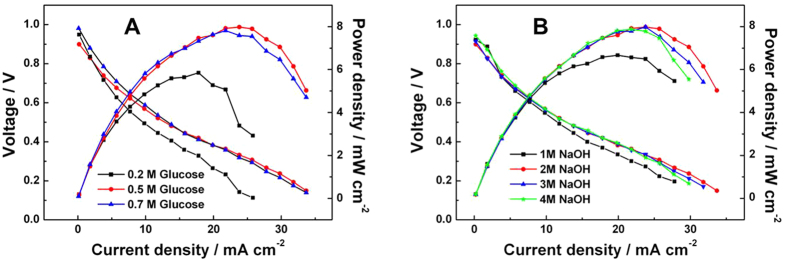
Single-cell performances of NPG-PtBi and Pt/C based MEAs. C-V and C-P polarization curves of DGFC in (**A**) 2 M NaOH with different glucose concentrations and (**B**) 0.5 M glucose with different NaOH concentrations. The anode NPG-PtBi loaded 0.15 mg_Pt_ cm^−2^ and 0.45 mg_Pt+Au_ cm^−2^, while the commercial Pt/C was used as cathode with Pt loading of 2 mg cm^−2^. The operation temperature was 60 °C.

The cell performance with different glucose concentrations at a NaOH concentration of 2 M is shown in Fig. 7A. The performance increases with glucose concentration from 0.2 to 0.5 M. However, further increase in glucose concentration to 0.7 M causes performance degradation. Therefore, there exists an optimal glucose concentration of 0.5 M with a peak power density of 8 mW cm^−2^. Considering the possible reasons, we suppose that the optimized glucose concentration is mainly due to competitive adsorption of glucose and hydroxyl, which are the two reactants of GOR in alkaline solutions. In addition, together with the increased glucose concentration, solution viscosity will be enhanced to hinder the mass diffusion, thus reduce the fuel cell performance.

The effect of NaOH concentration on cell performance is exhibited in Fig. 7B at a fixed glucose concentration of 0.5 M. The maximal power density increases from 6.5 to 8 mW cm^−2^ with the NaOH concentration increasing from 1 to 2 M because higher OH^−^ concentration can enhance the GOR kinetics^11^. However, the polarization curves gradually decreased when the NaOH concentration increasing from 2 to 3 and 4 M. The possible explanation for this phenomenon is the fewer and fewer adsorbed glucose molecules ascribed to the more and more adsorbed hydroxyl groups.

**Figure 8:**
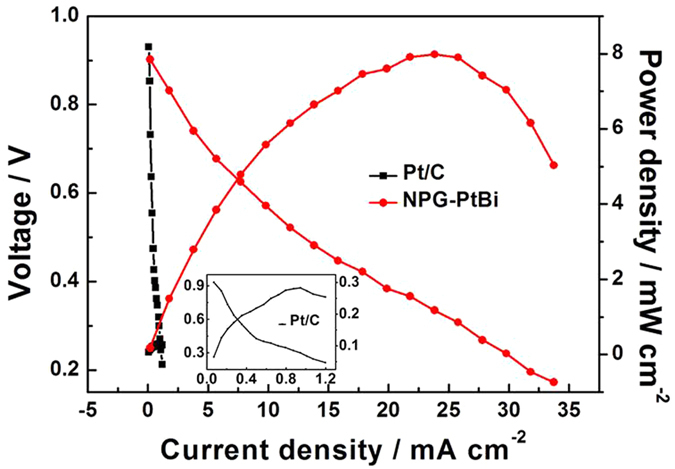
Single-cell performances of NPG-PtBi and Pt/C based MEAs. C-V and C-P polarization curves of DGFC separately using NPG-PtBi (0.15 mg_Pt_ cm^−2^ and 0.45 mg_Pt+Au_ cm^−2^) and commercial Pt/C (0.15 mg_Pt_ cm^−2^) as the anode. Commercial Pt/C was used as cathode with Pt loading of 2 mg cm^−2^. The test was operated in mixture solution of 2 M NaOH and 0.5 M glucose at 60 °C.

Finally, in comparation with standard electrocatalyst, commercial electrocatalyst JM-Pt/C (60%) was used as the anode in DGFC with the same Pt loading as NPG-PtBi. This MEA was constructed and tested in mixture solution of 2 M NaOH and 0.5 M glucose at 60 °C. As presented in Fig. 8, the peak power density of Pt/C is 0.24 mW cm^−2^. Comparing with the commercial Pt/C electrocatalyst, NPG-PtBi exhibits an outstanding performance, about 33-fold (53 mW mg_Pt_^−1^) when normalized to Pt loading. Even the power density is normalized to the total novel metal loading; 4.5 times enhancement could be obtained by using an NPG-PtBi anode (18 mW mg^−1^).

## Discussion

The results presented in this paper indicate that by using nanoporous gold films as substrates, we have successfully designed and fabricated a high performance anode electrocatalyst NPG-PtBi for DGFCs with ultra-low Pt loading. Unlike the other non-enzyme electrocatalysts for DGFC, NPG-PtBi anode possesses nanostructure but thin film in nature, which is easily fabricated and assembled for MEA. When compared with conventional powder anode catalysts, such as Pt/C and PtBi/C, NPG-PtBi anode can yield a much higher power output of 8 mW cm^−1^, which is almost 33 times higher than Pt/C catalyst. Therefore, including the enhanced hydroxyl adsorption from the synergetic effect of Bi, the high performance is mainly attributed to the bicontinuous ligament/pore structure of NPG substrate who offers accessible channels both for electron conduction and mass transfer. Furthermore, to decrease the utilization of noble metals, development of substitutes for NPG is on the agenda.

## Methods

### Preparation of NPG-PtBi

Au-Ag alloy films were purchased from Sepp Leaf Products, Inc. All chemical reagents were used as purchased without further purification. The NPG substrates were prepared by dealloying 100 nm-thick, 12-Karat Au-Ag alloy films in concentrated HNO_3_ (65%) at 30 °C for 30 min and then thoroughly rinsed in ultrapure water. Then, NPG-Pt was carried out by reducing H_2_PtCl_6 _using hydrazine hydrate vapor, as reported in previous papers^45^. The NPG films were first floated on the surface of 2 mM H_2_PtCl_6_ solution with pH value controlled at ~10, and then the reduction reaction happened in a confined space by using the vapor of hydrazine hydrate at 40 °C. Electro-deposition of Bi on NPG-Pt surface was operated at 0.03 V in mixture solution of 5 mM Bi^3+^ and 0.1 M HClO_4_^53^. A silver-silver chloride electrode and a carbon plate were used as reference and counter electrodes, respectively. Amounts of Bi specie were simply determined by controlling deposition times.

### Characterizations

Information about the morphology and composition of NPG-Pt, NPG-PtBi was obtained from SEM on an NOVA NanoSEM 230 equipped with Energy Dispersive X-ray Spectroscopy (FEI Corporate), TEM and HRTEM on a 200 kV JEM-2100 transmission electron microscope (JEOL Ltd.). The chemical state of Bi was analyzed with a Sigma Probe HA6000II (Thermo VG Scientific) X-ray photoelectron spectrometer, using Al Kα X-ray as the excitation source (1486.6 eV).

### Electrochemical measurements

The NPG-based electrodes were prepared by lift-coating glass carbon electrode (GCE) surfaces (ϕ = 4 mm) with the free-standing nanoporous electrocatalysts. Commercial Pt/C catalyst ink was prepared by dispersing 1 mg Pt/C (20 wt%) catalyst ultrasonically into a mixture of 0.5 ml Nafion alcohol solution (0.5 wt%) and 0.5 ml ultrapure (>18.23 MΩ cm) water for 10 minutes. 13 μL of the ink was drop-coated onto a GCE, resulting in a Pt loading of ~50 μg cm^−2^. The coating ink was dried for 20 min to form a uniform thin film on the GCE surface. Electrochemical measurements were carried out in a standard three-electrode cell, with the mercuric oxide electrode (Hg/HgO) and a Pt plate separately as reference and counter electrodes. All the electrolyte solutions were N_2_-saturated before electrochemical experiments. In comparison, cyclic voltammetric (CV) curves of each electrocatalyst were recorded using an electrochemical workstation (CHI 760D, CH Instruments, Inc., China) at a scan rate of 20 mV s^−1^ at 25 °C.

CO-stripping experiments were conducted in a conventional three compartment electrochemical cell using a 0.5 M H_2_SO_4_ electrolyte. A Pt plate counter electrode and saturated calomel electrode (SCE) reference electrode were used. Pure CO was bubbled through the electrolyte for 20 min and then the working electrode was immersed into this CO-saturated solution under potential control (0.14 V vs. RHE). After that, this solution was purged with N_2_ for 30 min to eliminate the dissolved CO. Finally, the adsorbed CO was oxidized in an anodic scan at 50 mV s^−1^.

### Single Fuel cell setup and operation

Firstly, a membrane electrode assembly (MEA) was constructed with a Nafion^®^ 115 membrane sandwiched between an anode and a cathode. The Nafion^®^ 115 membrane with a thickness of 127 μm, was produced by DuPont and purified to remove organic contaminants and heavy metal ions before utilization^43^. After purification, pieces of Nafion^®^ 115 membrane were immersed in 2 M NaOH for 12 h to obtain Na^+^ transfer property. Home-made anode NPG-PtBi film was directly attached to the carbon paper (TGP-H-060, Toray, Japan). Commercial Pt/C (60 wt%, Johnson Matthey, UK) was printed onto carbon paper as a cathode with a Pt loading of 2.0 mg cm^−2^. This sandwich structure was hot-pressed at 130 °C and 10 MPa for 3 minutes. As-prepared MEA was then fixed into a single cell test kit using graphite plates providing flow channels and collecting charges. The anodic fuel solution was fed into the anode flow channel by a peristaltic pump, while air was fed to the cathode through a mass flow controller. In addition, the cell temperature was controlled through a thermoelectric couple. Steady state polarization curves were recorded by using automatic electric load (PLZ 70UA, Japan). In comparison, commercial Pt/C catalyst was also tested as the anode with the same Pt loading to NPG-PtBi using the same processing technology as the cathode.

## Additional Information

**How to cite this article**: Guo, H. *et al*. Pt-Bi decorated nanoporous gold for high performance direct glucose fuel cell. *Sci. Rep.* **6**, 39162; doi: 10.1038/srep39162 (2016).

**Publisher’s note:** Springer Nature remains neutral with regard to jurisdictional claims in published maps and institutional affiliations.
